# Factors associated with breast cancer recurrences or mortality and dynamic prediction of death using history of cancer recurrences: the French E3N cohort

**DOI:** 10.1186/s12885-018-4076-4

**Published:** 2018-02-09

**Authors:** Alexandre Lafourcade, Mathilde His, Laura Baglietto, Marie-Christine Boutron-Ruault, Laure Dossus, Virginie Rondeau

**Affiliations:** 1 Research Center Inserm, U1219 Bordeaux, France; 2 University of Bordeaux, Bordeaux, France; 3 CESP Generations and Health Team, Paris-Saclay University, Paris-Sud Univ, UVSQ, INSERM, Villejuif, France; 4 Gustave Roussy, Villejuif, France; 5 Nutrition and Metabolism Section, International Agency for Research on Cancer, Lyon, France; 6 Biostatistic Team, INSERM U1219, University of Bordeaux, 146 rue Léo Saignat, CS 61292, F-33076 Bordeaux Cedex, France

**Keywords:** Breast cancer, Death, E3N cohort, Joint frailty model, Prognostic factors, Recurrences

## Abstract

**Background:**

In addition to tumor characteristics and lifestyle factors, cancer relapses are often related to the risk of death but have not been jointly studied. We investigate the prognostic factors of recurrent events and death after a diagnosis of breast cancer and predict individual deaths including a history of recurrences.

**Methods:**

The E3N (Etude Epidémiologique auprès de Femmes de la Mutuelle Générale de l’Education Nationale) study is a prospective cohort study that was initiated in 1990 to investigate factors associated with the most common types of cancer. Overall survival and three types of recurrent events were considered: locoregional recurrence, metastasis, and second primary breast cancer. Recurrent events and death were analyzed using a joint frailty model.

**Results:**

The analysis included 4926 women from the E3N cohort diagnosed with a first primary invasive breast cancer between June 1990 and June 2008; during the follow-up, 1334 cases had a recurrence (median time of follow-up is 7.2 years) and 469 women died. Cases with high grade, large tumor size, axillary nodal involvement, and negative estrogen and progesterone receptors had a higher risk of recurrence or death. Furthermore, smoking increased the risk of relapse. For cases with a medium risk profile in terms of tumor characteristics and lifestyle factors, the probability of dying between 5 and 10 years after diagnosis was 6, 20 and 36% for 0, 1 or 2 recurrences within the first 5 years after diagnosis, respectively.

**Conclusions:**

Our study showed the importance of considering baseline lifestyle characteristics and history of relapses to dynamically predict the risk of death in breast cancer cases. Medical experience coupled with an estimate of a patient’s survival probability that considers all available information for this patient would enable physicians to make better informed decisions regarding their actions and thus improve clinical output.

**Electronic supplementary material:**

The online version of this article (10.1186/s12885-018-4076-4) contains supplementary material, which is available to authorized users.

## Background

Breast cancer is the most common type of cancer in women worldwide and the leading cause of cancer death in women [1]. Between 8 and 10% of women diagnosed with breast cancer will present locoregional recurrences [2–4], and 15 to 30% will develop distant metastases [4].

Tumor characteristics are the main prognostic factors for breast cancer outcome. A tumor size larger than 2 cm, axillary nodal involvement, negative estrogen and progesterone receptors, and high grade have been shown to increase the risk of death after breast cancer diagnosis and the risk of locoregional recurrence and metastases [5]. Young age at diagnosis has also been associated with a worse clinical outcome, partly because of the over-representation of more aggressive subtypes in young cases compared to older cases, such as triple negative or HER2-positive breast cancer, or because of delayed diagnosis and presentation at an advanced stage [6].

Lifestyle factors might also influence the risk of recurrence and/or death after breast cancer diagnosis. Excess weight, which is associated with a higher risk of postmenopausal breast cancer, is also associated with a higher risk of breast cancer recurrence and death [7]. Smoking is weakly associated with breast cancer risk [8] but has been associated with a higher risk of death after breast cancer, with a 60% higher risk in current smokers at breast cancer diagnosis and a 50% risk in former smokers of over 35 pack-years [9, 10]. Similar findings have been reported for the risk of recurrence [10]. Alcohol intake, a risk factor for breast cancer, increases also the risk of recurrence [11, 12]. However studies investigating the interplay between alcohol and other important lifestyle risk factors and certain disease characteristics as well as genetic susceptibility are called for. In contrast, the relation between alcohol consumption and breast cancer survival is less clear [13].

Reproductive factors associated with breast cancer risk could influence breast cancer survival: women with a diagnosis of breast cancer before menopause have a greater risk of recurrence and death [3]. Although hormone replacement therapy (HRT) has been found to increase the risk of breast cancer in healthy women, pre-diagnostic HRT use showed no effect or a decreased mortality in most observational studies [14, 15]. Conversely, the Women Health Initiative (WHI) trial noted that mammary tumors diagnosed in the estrogen plus progestin (E + P) treatment group had worse prognostic characteristics compared to the placebo group [15].

Most studies on prognostic factors for breast cancer recurrence were performed using Cox proportional hazard models or competing-risks regression models [15]. In these studies, the follow-up of the patient ceased after the first relapse, and subsequent recurrences were not considered in the prediction of cancer-related death. The successive relapses can be analyzed using a shared frailty model for the recurrent events to account for the intra-subject correlation. However, in many settings, a joint modeling approach for time to recurrence and for death is required. First, in relation to the recurrences, given the strong relationship between recurrences and breast cancer-related death [3], death should not be considered an independent censoring process. Second, in relation to death, the number of recurrences occurring during follow-up should be considered a time-dependent covariate; in this context, the Cox proportional hazards model is not applicable because it requires that the time-dependent covariates be external (or exogenous), that is, their value at a specific time point *t* is not affected by the occurrence of an event at a previous time point [16].

To overcome these problems, Mauguen et al. [17] proposed to apply a joint frailty model [18, 19], that accounted both for the dependence between recurrences and the dependence between recurrences and death. They showed that when predicting the risk of death, accounting for relapses led to better prediction performance.

In this paper we applied a joint model to study the effects of tumor characteristics, lifestyle characteristics and reproductive factors on the risk of relapse and death after a first invasive breast cancer diagnosis using incident cases within the population-based E3N cohort. More specifically, we studied the dynamic prediction of the risk of death considering baseline lifestyle characteristics and history of recurrent events.

## Methods

### The E3N cohort

The E3N (Etude Epidémiologique auprès de Femmes de la Mutuelle Générale de l’Education Nationale) study is a prospective cohort study that was initiated in 1990 to investigate factors associated with the most common types of cancer [20]. It includes 98,995 women living in France who were born between 1925 and 1950 and were covered by a national health insurance plan that primarily covers teachers. The participants complete biennial self-administered follow-up questionnaires on health status, medical history and lifestyle. All of the subjects signed an informed consent form at study entry, and the study protocol was approved by the French National Commission for Computed Data and Individual Freedom. The E3N cohort is the French component of the European Prospective Investigation into Cancer and Nutrition (EPIC) and therefore the baseline questionnaire of E3N is based on the EPIC questionnaire. Detailed information in English on the EPIC study is available here: http://epic.iarc.fr/index.php.

### Ascertainment of recurrent events and death after breast cancer

The time-to-event was defined as the time between the date of diagnosis of the first invasive breast cancer and the date of each recurrent event or death. Three types of recurrent events were considered: locoregional recurrence, metastasis, and second primary breast cancer. Primary cancers and recurrent events were self-reported to the E3N team by the participants or their next-of-kin or were identified among the causes of death on the death certificate. The information was then validated via pathology reports or any other medical document. We then considered the date of the medical record as the exact date for the first breast cancer diagnosis and for the successive recurrent events. The participants’ vital status was regularly updated through the health insurance plan, postal service, and next-of-kin; causes of death were obtained from the French National Service on Causes of Death. In this work we considered all-cause mortality among breast cancer patients.

### Study population

The study population consisted of 5690 E3N women who were diagnosed with invasive non-metastatic breast cancer confirmed by medical records between June 1990 and June 2008. End of follow-up was defined as the date of death, date of last response to a questionnaire (censored), date of diagnosis of a recurrent event, or June 25, 2008, whichever occurred first. Women with in situ breast cancer (*N =* 735) or a metastatic breast cancer at first diagnosis (*N* = 29) were not included.

Among eligible cases, 242 women declared one or several recurrent events that were not confirmed and were then censored at the date of the event. The type of recurrent event was missing for 9 additional women; these events were removed and the women were censored at the time of the event. Thirty-five women died of breast cancer with metastases reported at death but not before. These events were imputed 2 years before death for 18 women (mean time between all types of relapse and death in this study) and 1 day before death for the others because the delay between cancer and death was less than 2 years.

### Statistical analyses

Recurrent events such as locoregional recurrence, metastasis, or second primary breast cancer may lead to death; conversely, because death prevents any new recurrent events, it is a competing event for recurrences, and these two processes may be correlated. Joint frailty models allow us to study the joint evolution over time of two survival processes by considering all successive recurrent events and the terminal event (death) [18, 19]. Prognostic factors considered are described in Tables 3 and 4 The characteristics of the first diagnosed tumor were considered: estrogen and progesterone receptor status, differentiation grade, tumor size, axillary nodal involvement. We also considered at the time of diagnosis: age, history of benign breast disease, history of diabetes, history of breast cancer in first-degree relatives, alcohol consumption and Body Mass Index (BMI). Other characteristics included the level of education, smoking status, menopausal status and use of menopausal hormonal treatment (MHT) in the year prior to the breast cancer diagnosis. We also adjusted for the smoking status after diagnosis because women may stop smoking after breast cancer diagnosis. The year of breast cancer diagnosis was used as a proxy for breast cancer care.

We proposed also a dynamic prediction tool to predict the risk of death over a certain period of time, given the history of relapses and the past history in terms of covariates for a patient. The estimated probability can be updated following a new disease relapse. Different scenarios were considered by varying the window of prediction and the number and timing of recurrent events as previously performed in Mauguen et al. [17]. Three different profiles of patients according to risk factors (low, medium and high risk profile) were considered. The analytical framework for the model and this dynamic prediction is provided in the (see Additional file 1). All of the analyses were performed using the Frailtypack 2.6 package of the R software [21, 22].

## Results

During follow-up, among the 4926 cases of the study sample, 549 patients (11.2%) were lost to follow-up, 1334 recurrent events occurred including 343 locoregional recurrences, 603 metastases, and 388 s primary breast cancers; 18% of women suffered at least one recurrent event, 12% suffered exactly one event, 4% suffered two events and 2.1% suffered three or more) (see Table 1).

**Table 1:** Statistical summary of number of events: *N* = 4926

	No.	%	Time between first cancer and event
			median [IQR]
Number of recurrent events by woman			
0	4040	82.0	
1	604	12.3	3.8 [1.8–7.2]
2	180	3.7	5.9 [3.3–9.2]
3–7	102	2.1	6.8 [3.8–9.5]
Type of event			
Local recurrence	343	25.7	3.9 [1.2–7.6]
Metastasis	603	45.2	4.8 [2.4–7.9]
Second primary breast cancer	388	29.1	5.4 [2.8–8.5]
All recurrences	1334	100.0	7.2 [3.9–11.2]
Death	469	9.5	5.6 [3.3–8.9]

*IQR* interquartile range

The median time between breast cancer and any recurrent event was 7.2 years (IQR: 3.9–11.2 years). The median time between breast cancer diagnosis and the first recurrence was 3.8 years for the first event, 5.9 years for the second event, and 6.8 years for the third event. Locoregional recurrences generally occurred sooner after breast cancer diagnosis (median = 3.9 years, IQR: 1.2–7.6) than metastases (median = 4.8 years, IQR: 2.4–7.9) or second primary breast cancers (median = 5.4 years, IQR: 2.8–8.5). Most events occurring after the first event were metastases (185 subjects, see Table 2).

**Table 2:** Mean time between two events in years and number of patients observed by type of event, until the 4th event (median follow-up time = 7.2 years)

	Cancer- 1st event		1st- 2nd event		2nd- 3rd event		3rd- 4th event		TOTAL	
End period event	Year (σ)	*N* (%)	Year (σ)	*N* (%)	Year (σ)	*N* (%)	Year (σ)	*N* (%)	Year (σ)	*N* (%)
Locoregional recurrence	4.1	276	3.4	47	1.8	6	2.2	7	4.9	343
	(3.8)	(5.6)	(3.3)	(5.3)	(2.5)	(2.1)	(2.7)	(7.1)	(3.6)	(5.5)
Metastasis	4.4	281	1.5	185	0.9	90	0.5	29	2.7	603
	(3.5)	(5.7)	(2.2)	(21.0)	(1.3)	(32.4)	(0.9)	(29.6)	(3.2)	(9.6)
Second primary breast cancer	5.6	329	3.4	50	2.6	6	2.3	2	5.3	388
	(4.1)	(6.6)	(3.4)	(5.7)	(3.6)	(2.1)	(3.1)	(2.0)	(4.0)	(6.2)
Death	5.3	179	2.5	128	1.9	91	1.3	44	3.3	469
	(3.3)	(3.6)	(2.3)	(14.6)	(1.9)	(32.9)	(1.4)	(44.9)	(3.0)	(7.5)
Censored	7.4	3861	5.1	464	3.3	84	2.3	16	7.1	4436
	(4.7)	(78.4)	(3.9)	(53.1)	(2.9)	(30.3)	(2.7)	(16.3)	(4.7)	(71.1)
TOTAL	7.0	4926	3.8	874	2.0	277	1.3	98	6.1	6239
	(4.7)	(100%)	(3.6)	(100%)	(2.3)	(100%)	(1.8)	(100%)	(4.7)	(100%)

σ = standard error

During follow-up, 469 women died. The median time between breast cancer and death was 5.6 years (IQR: 3.3–8.9 years). For the women who died, the time between the last non-fatal recurrent event and death decreased with the increasing number of recurrent events. In total, 4.4% of those with no recurrence, 20% of those with one recurrence, 50% of those with two recurrences and more than 70% of those with three or more recurrences died during follow-up.

We observed that 13% of the cases were double hormone receptor negative; 40% were poorly differentiated; 19% had a tumor larger than 2 cm and 28% had nodal involvement (see Table 3).

**Table 3:** Breast cancer characteristics of study population, *N* = 4926

	No. of women	Percentage
Estrogen/Progesterone receptors Status		
positive / positive	2321	47.1
positive / negative	770	15.6
negative / positive	161	3.2
negative / negative	636	12.9
missing	1038	21.1
Grade		
well differentiated	650	13.2
moderately differentiated	1517	30.8
poorly differentiated	1965	39.9
missing	794	16.1
Tumor size		
< 2 cm	3694	74.0
≥ 2 cm	956	18.5
missing	276	5.6
Axillary nodal involvement		
no	3233	65.6
yes	1378	27.9
missing	315	6.4
Year of diagnosis		
1990–1994	1010	20.5
1995–1999	1472	29.9
2000–2003	1321	26.8
2004–2008	1123	22.8

We observed that before the cancer diagnosis, 11% of the women were current smokers (see Table 4). After the cancer diagnosis, only 6% of the women continued to smoke.

**Table 4:** Characteristics of the study population, *N* = 4926

	No. of women	Percentage
Age at breast cancer diagnosis		
< 50 years	647	13.1
[50–60) years	2145	43.5
[60–70) years	1712	34.8
≥ 70 years	422	8.6
Menopausal status at breast cancer diagnosis		
postmenopausal woman - more than 5 years of cumulated MHT use	1144	23.2
postmenopausal woman - less than 5 years of cumulated MHT use	1205	24.5
postmenopausal woman - no use of MHT	1314	26.7
premenopausal woman	923	18.7
Missing	340	6.9
recent MHT use before diagnosis		
yes	1914	38.9
no	2932	59.5
missing	80	1.6
Smoking status before diagnosis		
never smoker	2624	53.3
current smoker	562	11.4
ex-smoker	1717	34.9
missing	23	0.5
Smoking status at first follow-up after diagnosis		
never smoker	2624	53.3
current smoker	314	6.4
ex-smoker	1664	33.8
missing	324	6,6
Family history of breast cancer before diagnosis		
0	3959	80.4
1	804	16.3
2–4	98	1.0
missing	65	1.3
History of benign breast disease before diagnosis		
Yes	2575	52.3
No	2351	47.7
History of diabetes before diagnosis		
Yes	258	5.2
No	4668	94.8
Level of education before diagnosis		
< high school level	568	11.5
high school level-second year university level	2351	47.7
> second year university level	1803	36.6
Missing	204	4.1
Alcohol consumption at diagnosis		
Yes	4105	83.3
no	523	10.6
Missing	298	6.0
BMI at diagnosis		
underweight (< 18.5)	132	2.7
normal (18.5- < 25)	3255	66.1
overweight (25- < 30)	1073	21.8
obese (≥ 30)	264	5.3
Missing	202	4.1

*MHT* menopausal hormonal treatment, *BMI* Body Mass Index

Table 5 presents the results of the multivariate joint frailty model. Negative hormonal receptors, poorly differentiated tumor, large tumor size and nodal involvement were all associated with an increased risk of recurrent events and death. The recent use of MHT was associated with a decreased risk of recurrence (hazard ratio (HR) = 0.75, 95% CI: 0.62, 0.92) but was not associated with the risk of death. Smoking increased the risk of relapse (HR for current smoker vs never smoker = 1.55, 95% CI: 1.16, 2.07). Breast cancers diagnosed after 2000 were significantly less likely to relapse than those diagnosed before. This result is likely linked to the shorter follow-up time for those with a diagnosis after 2000 and thus a lower probability to observe recurrences. The risk of death was higher when breast cancer was diagnosed after age 60 (HR = 4.50, 95% CI: 1.29, 15.78 for 60–70 vs < 50 years and HR = 9.08, 95% CI: 1.89, 43.69 for 70+ vs < 50 years). Menopausal status was not a significant risk predictor.

**Table 5:** Joint model estimation on population (*N* = 2708, 632 recurrent events, 212 deaths)

Variables (% of women)	Recurrent event		Overall death	
	HR	95% CI	HR	95% CI
Estrogen/Progesterone receptor status				
+/− vs +/+ (18.6%)	1.40	1.10, 1.79	2.51	1.20, 5.30
−/+ vs +/+ (4.1%)	1.00	0.62, 1.59	5.63	1.55, 20.46
−/− vs +/+ (15.6%)	2.14	1.70, 2.70	9.76	4.39, 21.75
Grade				
moderately differentiated vs well differentiated (36.4%)	1.61	1.15, 2.27	3.12	0.99, 9.91
poorly differentiated vs well differentiated (48.5%)	1.90	1.36, 2.66	6.54	2.02, 21.18
Tumor size (≥ 2 cm vs < 2 cm) (20.5%)	1.64	1.33, 2.01	4.70	2.27, 9.75
Axillary nodal involvement (yes vs no) (30.0%)	1.54	1.26, 1.86	5.23	2.63, 10.42
Recent MHT use before diagnosis (yes vs no) (44.3%)	0.75	0.62, 0.92		
Smoking status first follow-up after diagnosis				
ex-smoker vs never smoker (36.9%)	1.20	1.00, 1.44		
current smoker vs never smoker (6.5%)	1.55	1.16, 2.07		
Year of diagnosis				
1995–1999 vs 1990–1994 (28.9%)	0.90	0.72, 1.13		
2000–2003 vs 1990–1994 (32.3%)	0.72	0.56,0.94		
2004–2008 vs 1990–1994 (27.4%)	0.61	0.43, 0.89		
Age at breast cancer diagnosis (year)				
[50–60) vs < 50 (44.1%)			1.80	0.68, 4.79
[60–70) vs < 50 (38.0%)			4.50	1.29, 15.78
≥ 70 vs < 50 (9.5%)			9.08	1.89,43.69
Menopausal status at breast cancer diagnosis				
postmenopausal woman - no use of MHT vs premenopausal woman (27.1%)			1.39	0.58, 3.32
postmenopausal woman-less than 5 years of cumulated MHT use vs premenopausal woman (29.3%)			0.79	0.32, 1.97
postmenopausal woman-more than 5 years of cumulated MHT use vs premenopausal woman (28.8%)			0.35	0.11, 1.10
θ	1.01 (σ = 0.05) *p*-value< 0.001			
α	4.29 (σ = 0.77) *p*-value< 0.001			

*HR* hazard ratio, *σ* standard error, *95% CI* 95% Confidence Interval, *MHT* menopausal hormonal treatment

The risk of death was strongly dependent on the history of recurrence even after adjusting for the covariates (frailty variance θ and power α significantly different from zero).

### Dynamic prediction of the risk of death

**Fig. 1:**
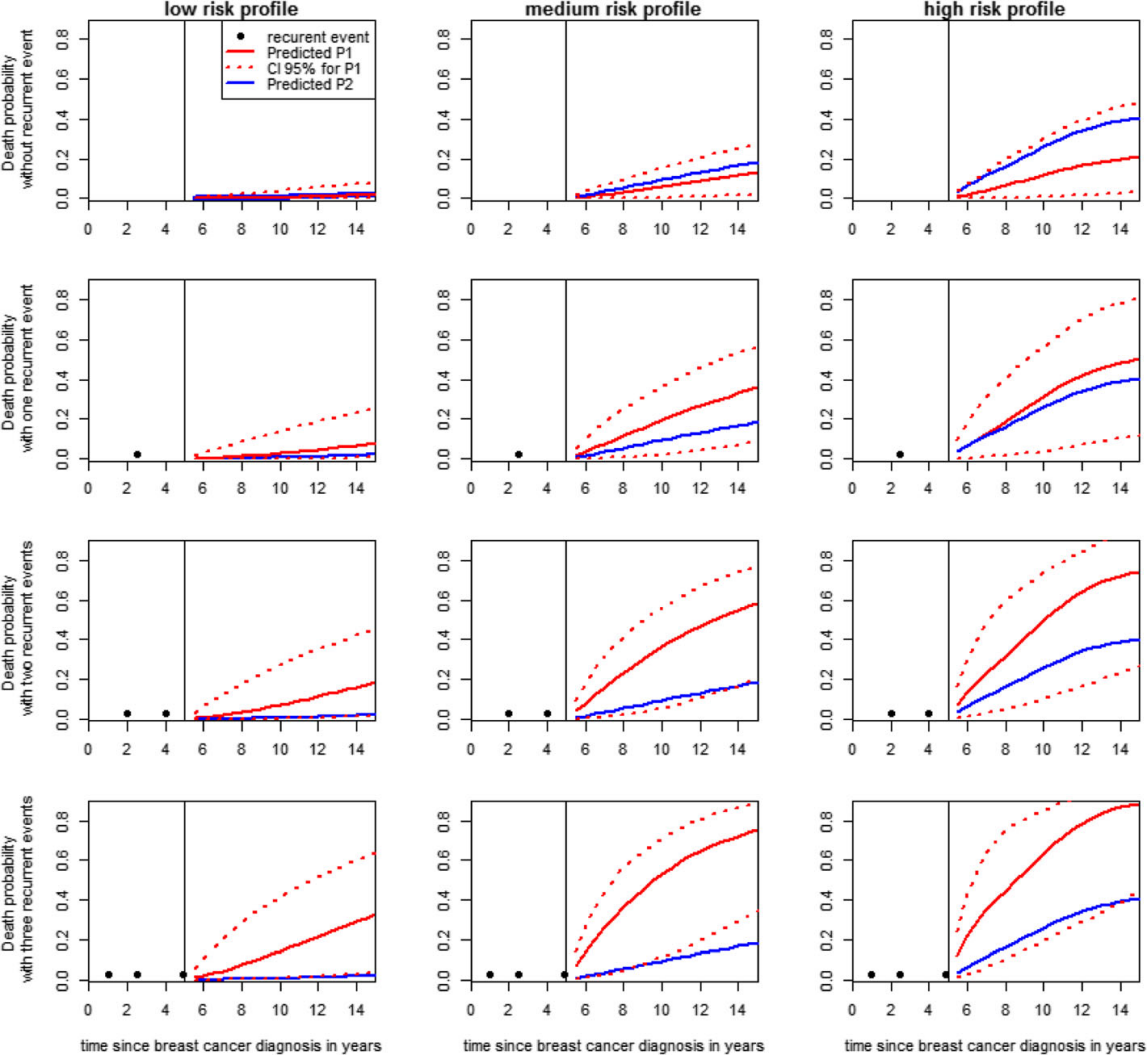
Individual predicted probabilities of death for 3 patient profiles, with or without a history of relapses using joint frailty models. ***P*1**: probability of death considering exactly J recurrent events, ***P*2**: probability of death not considering the history of recurrent events. **Low-risk profile**: Estrogen/Progesterone receptor status = +/+, Grade = 1, Tumor size≤2 cm, Axillary nodal involvement = no, recent MHT use before diagnosis = yes, Smoking status = never smoker, Year of diagnosis = 2004–2008, Age at breast cancer diagnosis≤50 years, Menopausal status = postmenopausal woman - less than 5 years of cumulated MHT use. **Medium-risk profile**: Estrogen/Progesterone receptor status = +/−, Grade = 2, Tumor size≥2 cm, Axillary nodal involvement = no, recent MHT use before diagnosis = yes, Smoking status = ex-smoker, Year of diagnosis = 1995–1999, Age at breast cancer diagnosis = [50–60) years, Menopausal status = premenopausal woman. **High-risk profile**: Estrogen/Progesterone receptor status = −/−, Grade = 3–4, Tumor size≥2 cm, Axillary nodal involvement = yes, recent MHT use before diagnosis = no, Smoking status = smoker, Year of diagnosis = 1990–1994, Age at breast cancer diagnosis≥70 years, Menopausal status = postmenopausal woman - no use of MHT

Individual dynamic prediction of death was performed on three profiles of risk factors identified on the basis of the previous results: a low, medium, high-risk profile (see Fig. 1). When the history of recurrent events was not considered (P2), the probability of death was overestimated if no recurrent events were diagnosed and underestimated if recurrent events occurred.

The number of recurrent events greatly affected the risk of death: for the medium risk profile, the probability of death (P1) between 5 and 10 years after cancer diagnosis was 6% (95% CI: 1, 16) for no recurrence in the first 5 years after diagnosis, 19% (95% CI: 2, 37) for one recurrence, 36% (95% CI: 5, 56) for two recurrences and 53% (95% CI: 11, 70) three previous recurrences; between 5 and 15 years, the risk of death was 13% (95% CI: 3, 28) if no recurrent events occurred and 36% (95% CI: 10, 57), 59% (95% CI: 23, 78) and 76% (95% CI; 38, 89) if one, two or three events occurred, respectively. The risk of death was also affected by risk factors: after two recurrent events, the risk of death between 5 and 10 years after the cancer diagnosis was 7, 36 and 51% in women with low-, medium-, and high-risk profiles, respectively.

To estimate whether the predictions are accurate, error of prediction curves based on the brier score were used. Prediction errors (not presented here) were influenced by the number and timing of relapses. Indeed, with a Cox model, the prediction error of the risk of death between 5 and 10 years was 9%, whereas in the joint frailty model, which accounts for relapse times, the prediction error was 8% with probability P2 and 7% with probability P1.

## Discussion

In this article, we studied jointly the prognostic factors for successive relapses and death on a large cohort of women with a primary breast cancer. We proposed a dynamic individual prediction of the risk of death after breast cancer using a history of cancer recurrences, tumor characteristics, lifestyle characteristics and reproductive factors, showing that at a given time after diagnosis, the risk of death was strongly dependent on the number of relapses.

To date, relatively few studies have attempted to study this association. Our results confirm the relevance of the association between established prognostic factors, MTH and cigarette smoking and the risk of recurrent events or death after breast cancer. The association between lifetime cigarette smoking and risk of breast cancer recurrence has been previously described into the literature [10]. The tumor characteristics (high grade, high tumor size, nodal involvement) were also associated with a higher risk of those two failure times, as previously shown by Mauguen et al. [17]. Conversely, the risk of relapses or death was lower for tumors with positive estrogen or progesterone receptors [23]. We observed as in previous observational studies [24], a lower risk of relapse for postmenopausal women who had used MHT before breast cancer diagnosis. However, women on MHT undergo more frequent mammographic monitoring and thus have an earlier cancer diagnosis [15]. Therefore, the observed better prognosis in terms of relapses for women using MHT in our study is possibly biased. However, we adjusted for the mammographic monitoring as a binary time dependent covariate (with at least one mammographic visit before the time of interest or without mammography). This variable was non-significantly associated with both the risk of recurrence (HR = 1.25, 95% CI: 0.98, 1.59) and the risk of death (HR = 0.62, 95% CI: 0.27, 1.46). Furthermore, this adjustment did not change the effect of the MHT use. Menopausal status (with or without history of MHT use) was not significant in the joint frailty model, even if the same tendencies were observed with the use of MHT.

Smoking exposure after breast cancer diagnosis was associated with an increased risk of relapse but not with the risk of death. This might be explained by the fact that almost half of the smokers stopped smoking after their cancer diagnosis. Obesity, a factor that has been previously associated with the risk of recurrence and death after breast cancer [7], was not significant in our study. However, E3N women are relatively slim (less than 6% were obese at breast cancer diagnosis), which might have resulted in a limited power to detect an association in this subgroup. Our study did not confirm any association of alcohol consumption or education level and the risk of recurrences or death. The year of breast cancer diagnosis was used as a proxy for breast cancer care. We did not obtain a significant association with this year of diagnosis and the risk death however the risk of recurrence was significantly lower after 2000. This might probably be explained by three reasons. Due to the design of the cohort, the breast cancer diagnosed in the 1990’ties will have longer follow-up than breast cancer diagnosed more recently. We also completed these results by a sensitivity analysis on the same sample but more homogeneous in terms of follow-up durations. In this new sample, the follow-up of the subjects was truncated at 10 years and those with a follow-up greater than 10 years were censored at 10 years. The results, on these patients with shorter follow-up times showed really similar findings, with the same association on the risk of recurrence and on death. Furthermore the introduction of Trastuzamab (Herceptin) in 1998 for the adjuvant treatment of Her2+ breast cancer has significantly reduced relapse rates. Finally since 2004 in France, there is a countryside breast cancer screening program for women aged 50–74. This recent screening program is associated with breast cancers diagnosed earlier and at an earlier stage. This can also explain the reduction of recurrence rates with time. However adjusting also for the mammography monitoring as a binary time dependent covariate did not change the associations.

A major limitation of this study is the absence of variables describing the cancer treatments. The year of breast cancer diagnosis, in combination with tumor characteristics, can be viewed as a proxy variable linked to the treatment evolution. The results reflect a lower risk of relapses for those recently diagnosed who are likely using aromatase inhibitors and practicing the technique of sentinel lymph node. However, for patients diagnosed after 1999, the follow-up time is shorter and the probability of observing a relapse is consequently lower.

We have illustrated that some specific profiles of breast cancer patients vary according to tumor characteristics, lifestyle characteristics, reproductive factors and the history of relapses; these factors may predict different probabilities of dying in the future. The proposed approach was able to show that the number of relapses greatly affects the predicted risk of death and that better predictions of death are obtained by considering the history of relapses rather than only considering prognostic factors. For example, the “naïve” probability of death that does not consider the history of relapses was overestimated. Using this individual dynamic prediction tool, we illustrated that death probabilities must be updated each time additional relapses are recorded. In our analysis, we did not consider time-dependent covariates, such as modifications in the patient’s treatment, BMI, smoking habits or MHT use. However, similar to how these time-dependent covariates are allowed in the joint modeling, they are allowed in the prediction calculation. Although all of the analyses were performed using a standard statistical R package, a level of expertise in programming may be required. An easy web application as Adjuvant Online [25] could provide the opportunity for every physician to derive updated predictions for new patients when history of relapses or more outcomes (such as longitudinal biomarkers) are available, as in Krol et al. [26].

## Conclusion

In conclusion, survival outcomes after a breast cancer are affected by the occurrence of relapses, and the proposed approach (with joint model) is really appropriate to both study their link and to predict the prognosis of patients suffering from a primary breast cancer and with possible relapses. These dynamic prediction tools would be valuable in everyday medical practice. That is, medical experience coupled with an estimate of a patient’s survival probability that considers all available information for this patient would enable physicians to make better informed decisions regarding their actions and patient care and thus improve clinical output.

## Additional file

Additional file 1: Joint frailty model and prediction (DOCX 27 kb)

## Abbreviations

BMI: Body Mass Index
E3N: Etude Epidémiologique auprès de Femmes de la Mutuelle Générale de l’Education Nationale
HER2: Human Epidermal growth factor *Receptor* 2
HR: Hazard Ratio
HRT: Hormone Replacement Therapy
IQR: Inter Quartile Range
MHT: Menopausal Hormonal Treatment
WHI: Women Health Initiative
