# Supplementary material for: Factors associated with breast cancer recurrences or mortality and dynamic prediction of death using history of cancer recurrences: the French E3N cohort
Source: BMC Cancer. 2018 Feb 9;18:171. doi: 10.1186/s12885-018-4076-4 (PMC5807734; doi:10.1186/s12885-018-4076-4)
Supplement: Supplementary file 1 — Joint frailty model and prediction (DOCX 27 kb) [file 12885_2018_4076_MOESM1_ESM.docx]

**Additional file 1: Joint frailty model and prediction**

For subject i (i=1,…,N), we denote the following: X_ij_ is the j^th^ recurrent time (j=1,…,n_i_), C_i_ is the censoring time (not death), and D_i_ is the death time. T_ij_^R^=min (X_ij_,C_i_,D_i_) corresponds to each follow-up time, and δ_ij_^R^ is a binary indicator for recurrent events, which is 0 if the observation is censored or if the subject died and 1 if X_ij_ is observed (δ_ij_^R^ = I[T_ij_^R^ =X_ij_]) where I[] denotes indicator function). Similarly, we denote T_i_^D^ as the last follow-up time for subject i, which is either a time of censoring or a time of death (T_i_^D^=min(C_i_,D_i_)) and δ_i_^D^ = I[T_i_^D^ =D_i_]). We observe the sequence (T_ij_^R^, δ_ij_^R^, T_i_^D^, δ_i_^D^). Finally, we denote Z_ij_^R^ and Z_i_^D^ as the vectors of covariates associated with the risk of recurrent events and death, respectively. Both death and recurrent times are in the calendar timescale, that is, they are measured by the time elapsed since the origin of the study (breast cancer diagnosis). However, a patient is considered at risk of a j^th^ recurrence only after the (j-1)^st^ recurrence. The joint frailty models (18) enabled dependence between repeated and terminal event processes via a common term called “frailty” as follows:

$$\left\{ \begin{aligned} \lambda_{ij}^{R}\left( t | u_{i} \right)=u_{i}\lambda_{0}^{R}(t)\exp\left( \beta_{1}^{'}Z_{ij}^{R} \right) \text{(all recurrent events)} \\ \lambda_{i}^{D}\left( t | u_{i} \right)=u_{i}^{\alpha}\lambda_{0}^{D}(t)\exp\left( \beta_{2}^{'}Z_{i}^{D} \right) \text{(death)} \end{aligned} \right.$$

(1)

$$\text{with} E(u_{i})=1 var(u_{i})=\theta u_{i}\sim\Gamma\left( \frac{1}{\theta},\frac{1}{\theta} \right)$$

in which $\lambda_{0}^{R}\left( t \right)$ and $\lambda_{0}^{D}\left( t \right)$ are the baseline hazard functions for the risk of recurrence and the risk of death, respectively. The effects of the explanatory variables β_1_ and β_2_ are assumed to be different for the risk of recurrent events and the risk of death. The recurrent and death events are linked by the patient-specific frailty effect $u_{i}$ (independent, identically and gamma distributed). The between-subject heterogeneity is considered significant if the variance of the frailty $\theta$ differs from 0. The presence of the α term allows more flexibility in the model. When α=1, the frailty has an identical effect on the risk of recurrent events and on the risk of the terminal event. When α>0, the recurrent events rate and the terminal event rate are positively associated. Finally, α=0 would show that $\lambda_{i}^{D}\left( t | ui \right)$ does not depend on $u_{i}$ and, thus, that the terminal event process does not depend on the recurrent events process. The interpretation of α is logical only when the variance α is significantly different from zero.

Variable selection was performed through backward selection with a Cox regression model for death and with a shared frailty model (25) for recurrences: in the backward selection, all prognostic variables (described in the “statistical analysis” section) with a significance level of the Wald test higher that 0.20 were excluded; the selection procedure ceased when all of the variables left in the multivariate model were significant at a 5% threshold. All of the variables retained were included in the joint frailty model. The "smoking status before cancer diagnosis" variable was excluded because it was highly correlated with the "smoking status after cancer diagnosis" variable.

We proposed a dynamic prediction of the risk of death between a prediction time t and a prediction horizon *t + w* considering information available at time t, including baseline covariates and history of recurrent events (locoregional relapse or distant metastasis) until time t. The joint frailty model was used to make those predictions under different scenarios by varying the window of prediction and the number and timing of recurrent events as previously performed in Mauguen et al. (17). Let *t* and *w* be the time of prediction and the window of prediction, respectively. Consider a new subject i who is alive at time t, for whom we observe *J* recurrences before time *t* (i.e., we observe *X_i1_* < X_i2_ < …< X_iJ_ *<* *t*) and for whom the vectors of the covariates $Z_{ij}^{R}$ relative to the risk of recurrences and $Z_{i}^{D}$ relative to the risk of death are available at time t of prediction. Two probabilities of death were defined. The first was the probability of death between *t* and *t + w* considering J recurrent events (P1). The second was the probability of death between *t* and *t + w* considering the recurrent history only in the parameter estimation but not considering the history of recurrent events in the probability of death (P2). The quality of the joint frailty model predictions were compared to the Cox model by a Brier score. The technique of ten-fold cross-validation described by Mauguen and al. was used (17).
